# Therapeutic Magnetic Fields in Oncology: A Systematic Review of Safety and Supportive Clinical Use

**DOI:** 10.7759/cureus.101970

**Published:** 2026-01-21

**Authors:** Saverio Colonna, Fabio Casacci

**Affiliations:** 1 Rehabilitation Medicine, Spine Center, Bologna, ITA; 2 Research and Development, Osteopathic Spine Center Education, Bologna, ITA; 3 Rehabilitation, Spine Center, Bologna, ITA

**Keywords:** cancer supportive care, chemotherapy-induced peripheral neuropathy, integrative oncology, magnetic field therapy, musculoskeletal comorbidities, oncological safety, pulsed electromagnetic fields (pemf), quality of life, rehabilitation in cancer patients, systematic review

## Abstract

Therapeutic magnetic fields are widely used in rehabilitation and physiotherapy for musculoskeletal and neurological conditions. However, their use in oncological patients has historically been approached with caution and is often considered contraindicated, despite limited clinical evidence supporting this concern.

A systematic review was conducted in accordance with PRISMA guidelines. PubMed/MEDLINE was searched for studies evaluating therapeutic magnetic fields in oncological populations. Studies were classified according to predefined research questions addressing oncological safety, supportive care during cancer treatment, and management of comorbidities. Study selection, data extraction, and qualitative synthesis were performed.

A total of 3,841 records were identified. Only one randomized controlled trial specifically assessed oncological safety outcomes and found no evidence of disease progression, reduced survival, or impaired treatment response associated with magnetic field exposure. A larger body of heterogeneous evidence evaluated magnetic fields as supportive interventions during oncological treatments, particularly for chemotherapy-induced peripheral neuropathy, quality of life, and symptom burden. These studies reported improvements in neurotoxicity, patient-reported outcomes, and treatment tolerability, although methodological limitations were common. Evidence regarding pain management and rehabilitation in oncological remission was predominantly observational.

Current evidence does not support the assumption that therapeutic magnetic fields are inherently harmful in oncological patients. While data on oncological safety remain limited, available studies do not indicate adverse oncological outcomes. Magnetic field therapy may represent a feasible supportive intervention for symptom management and rehabilitation in selected oncological populations. However, well-designed prospective trials are required to better define safety profiles, clinical efficacy, and appropriate indications.

## Introduction and background

Low-intensity and/or low-frequency magnetic fields have been used for decades in rehabilitation and physiotherapy for the treatment of pain and musculoskeletal disorders.

From a physical standpoint, magnetic fields can be distinguished into static and dynamic fields and classified according to their intensity and frequency [1]. Based on intensity, magnetic fields are categorized as weak (<1 mT), medium-intensity (1 mT-1 T), and high-intensity (>1 T) fields. According to frequency, they can be classified as low-frequency magnetic fields (<30 kHz), radiofrequency fields (30-300 kHz), intermediate-frequency fields (300 kHz-3 MHz), and high-frequency fields (>3 MHz) [1].

Magnetic fields used for therapeutic and rehabilitative purposes generally fall within the low-intensity and low-frequency categories and are associated with non-ionizing radiation [2]. Unlike high-frequency ionizing radiation, which is known for its ability to induce direct DNA damage and for its established oncogenic role, low-frequency magnetic fields act predominantly through non-thermal mechanisms, modulating biochemical reactions and cellular processes without inducing ionization [2]. This distinction is particularly relevant in the oncological context, as it allows a conceptual separation between therapeutic exposure to magnetic fields and forms of radiation for which there is consolidated evidence of oncogenic risk.

The application of pulsed electromagnetic fields (PEMFs) represents a non-invasive modality that, in several clinical conditions such as osteoarthritis [3] and chronic low back pain [4-6], has been shown to reduce pain intensity and improve function compared with sham controls or standard treatments [7].

Studies on hip and knee osteoarthritis report that the application of PEMFs is associated with reductions in pain and joint stiffness, as well as improvements in physical function, with potential benefits for patients’ quality of life [8]. In addition, evidence from randomized clinical trials indicates that the addition of PEMFs to conventional physiotherapy protocols may lead to a decrease in pain intensity and a reduction in the use of analgesic medications in musculoskeletal pain disorders [9]. Although heterogeneity persists in treatment protocols and applied parameters, these findings support the notion that the use of low-frequency magnetic fields is clinically widespread and potentially effective in modulating pain and improving function in musculoskeletal disorders [4].

It is also important to consider that many oncological patients present with musculoskeletal comorbidities commonly observed in the general population, such as low back pain and osteoarthritis, which may significantly contribute to disability and reduced quality of life. As previously reported, in this context, several clinical studies have demonstrated the effectiveness of PEMFs in improving pain and function in patients with low back pain [4-6] and osteoarthritis [7]. Therefore, in the absence of evidence indicating oncological harm, the potential use of such physical therapies for the treatment of musculoskeletal comorbidities in oncological patients warrants consideration and should be evaluated on a case-by-case basis.

Although the mechanisms of action of magnetic fields are still under investigation, and protocol standardization has not yet been clearly established, available clinical evidence suggests a potential benefit in pain modulation and functional improvement in musculoskeletal disorders. Nevertheless, the methodological quality and heterogeneity of the studies require cautious interpretation.

The intrinsic limitations of conventional oncological therapies, including systemic toxicity [10], the development of pharmacological resistance, and restricted tissue penetration in specific anatomical districts, have stimulated interest in alternative or complementary therapeutic and supportive approaches [11,12]. In this context, several non-invasive physical modalities, such as light, electric fields, ultrasound, and magnetic fields, have received increasing scientific attention. Magnetic fields, in particular, are characterized by a high capacity for tissue penetration and by the absence of ionizing effects [10,13], clearly distinguishing them from ionizing radiation, which represents one of the few well-established oncogenic risk factors. Although exposure to extremely low-frequency electromagnetic fields was previously classified as “possibly carcinogenic” based on epidemiological studies [14,15], subsequent evaluations have not confirmed evidence of long-term harmful effects [16,17].

In the clinical setting, exploratory studies have also been published evaluating prolonged exposure to low-intensity electromagnetic fields in patients with advanced cancer. In an uncontrolled phase I/II study involving patients with advanced hepatocellular carcinoma, the application of radiofrequency electromagnetic fields modulated at tumor-specific frequencies was well tolerated, with no evidence of significant toxicity or accelerated disease progression, albeit in the absence of a control group [18].

In parallel with the clinical literature, several preclinical [19-21] and translational studies have investigated the interaction between electromagnetic fields and tumor cells, focusing on biological mechanisms such as proliferation, apoptosis, and modulation of signaling pathways. While these studies are useful for understanding biological plausibility and potential theoretical concerns, they remain confined to the experimental setting and do not allow direct inferences regarding clinical safety or efficacy in oncological patients.

Despite the widespread clinical use of therapeutic magnetic fields in rehabilitation and supportive care, their application in oncological patients remains controversial and is often discouraged in clinical practice, particularly in the presence of active or metastatic disease [22]. This cautious approach appears to be largely driven by theoretical concerns and precautionary principles rather than by robust clinical evidence demonstrating oncological harm [15].

While several experimental and preclinical studies have explored the biological interaction between low-frequency magnetic fields and tumor-related processes, clinical data specifically addressing oncological safety outcomes remain scarce and fragmented [23]. To date, no systematic review has specifically focused on evaluating whether therapeutic magnetic field exposure in oncological patients is associated with adverse oncological outcomes, such as disease progression, recurrence, or reduced survival.

Accordingly, the present systematic review was designed to address this gap by critically appraising the available clinical evidence on the oncological safety of therapeutic magnetic fields, while also exploring their potential role as supportive interventions and in the management of comorbidities in oncological patients.

In light of these considerations, a systematic evaluation of the available literature is warranted. The aim of this systematic review is threefold: (1) to analyze whether therapeutic exposure to low-intensity and/or low-frequency magnetic fields in oncological patients is associated with adverse oncological outcomes, such as disease progression, recurrence, or reduced survival; (2) to evaluate the potential role of magnetic fields as a supportive or adjunctive intervention, in association with conventional oncological therapies, in improving disease- or treatment-related symptoms without a direct antineoplastic intent; and (3) to explore the effectiveness of magnetic fields in the management of comorbidities in oncological patients, such as osteoarthritis and neuropathies, with the aim of improving quality of life.

## Review

Materials and methods

Study Objectives

In light of the widespread clinical use of magnetotherapy in rehabilitation settings, and the persistent caution regarding its application in oncological patients, and in accordance with the objectives defined in the Introduction, this systematic review aims to critically evaluate the available evidence according to three main objectives.

The primary objective (Q1) of the study is to assess whether therapeutic exposure to low-intensity and/or low-frequency magnetic fields in patients with active or previous malignancy is associated with adverse oncological outcomes, such as disease progression, recurrence, metastasis, or reduced survival.

The secondary objective (Q2) is to explore, in the absence of evidence of oncological harm, the potential clinical applicability of magnetic fields for the treatment of musculoskeletal comorbidities commonly observed in oncological patients, such as low back pain and osteoarthritis, based on evidence of efficacy available in the general population.

The tertiary objective (Q3) is to analyze the role of magnetic fields as a supportive or adjunctive intervention during conventional oncological therapies (chemotherapy, radiotherapy, or other treatments), with particular attention to improvements in quality of life, symptom reduction, and treatment tolerability, while simultaneously verifying the absence of negative effects on oncological outcomes.

Search Strategy

A comprehensive electronic search was conducted using a predefined search strategy. In addition, supplementary targeted searches were performed to identify relevant studies not retrieved through the primary search, including screening the reference lists of included articles, citation tracking of key papers, and targeted searches on specific clinical or preclinical topics relevant to the research questions. These additional searches were undertaken to ensure the completeness of the evidence base and to capture pertinent studies addressing oncological safety, supportive care, or the management of comorbidities.

This systematic review was conducted and reported in accordance with the Preferred Reporting Items for Systematic Reviews and Meta-Analyses (PRISMA) guidelines [24]. A comprehensive electronic literature search was conducted using PubMed/MEDLINE as the primary database. To enhance the completeness and sensitivity of the search, Scopus and Web of Science were also searched. In addition, the Cochrane Library was screened to identify existing systematic reviews or randomized controlled trials relevant to the research questions. Additional targeted searches, including screening of reference lists and citation tracking, were conducted to ensure completeness. The detailed search strategy and eligibility criteria are summarized in Table 1.

**Table 1:** Literature search strategy and eligibility criteria.

Component	Details
Databases searched	PubMed/MEDLINE
Time frame	From inception to December 2025
Search terms	("Magnetic Fields"[MeSH] OR magnetotherap* OR PEMF OR "pulsed electromagnetic field*" OR "low-frequency magnetic field*") AND ("Neoplasms"[MeSH] OR cancer* OR oncolog* OR tumor* OR tumour* OR malignan*)
Boolean operators	AND, OR
Filters applied	Human studies
Language	No language restrictions at the search level; inclusion is limited to studies with accessible full text
Supplementary search	Manual screening of reference lists and citation tracking of key articles
Exclusion criteria	Preclinical-only studies (in vitro or animal models without clinical data); non-therapeutic exposure studies; studies on environmental, residential, or occupational magnetic field exposure (e.g., power lines, transformer stations); non-oncological populations; lack of clinically relevant outcomes

The full electronic search string applied in PubMed/MEDLINE is reported below to ensure transparency and reproducibility of the search strategy.

("Magnetic Fields"[Mesh] OR magnetotherap*[tiab] OR "pulsed electromagnetic field*"[tiab] OR PEMF[tiab] OR "low-frequency magnetic field*"[tiab] OR "static magnetic field*"[tiab]) AND ("Neoplasms"[Mesh] OR cancer*[tiab] OR oncolog*[tiab] OR tumor*[tiab] OR tumour*[tiab] OR malignan*[tiab]).

PRISMA Flow - Methods Description

**Figure 1:**
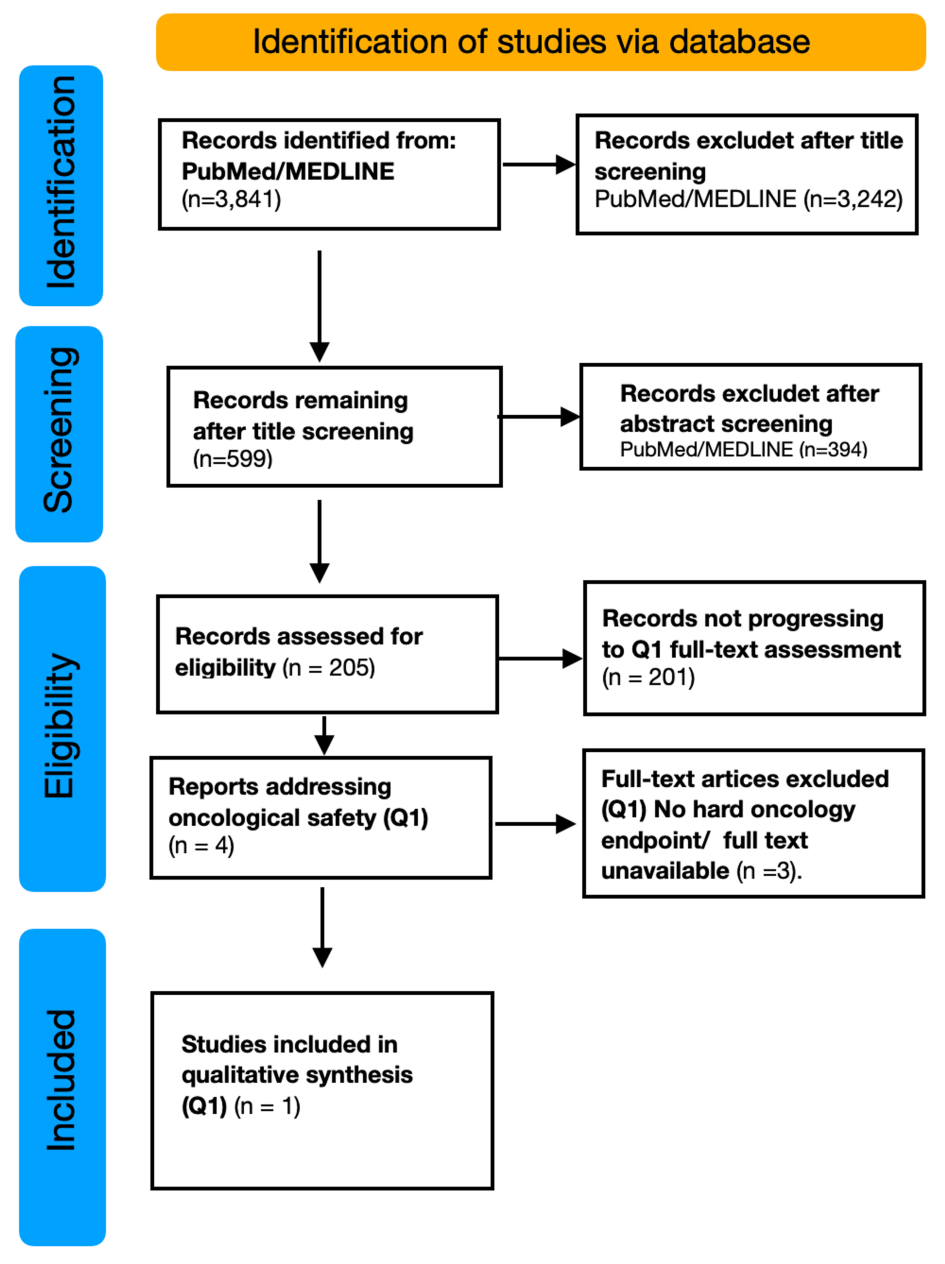
PRISMA flow diagram of the study selection process. Records were identified through PubMed/MEDLINE database searching and screened by title and abstract. After abstract screening, reports assessed for eligibility were classified according to the predefined research questions (Q1-Q3). Only studies addressing oncological safety (Q1) were assessed at the full-text level and included in the qualitative synthesis. Reasons for exclusion at each stage are reported. Image credit: Saverio Colonna

A comprehensive electronic search was conducted in PubMed/MEDLINE using a predefined search strategy combining terms related to therapeutic magnetic fields and oncology. The search yielded 3,841 records. Supplementary targeted searches were performed through reference list screening and focused searches to identify additional relevant studies (Figure 1).

Deduplication: All records retrieved from PubMed were imported into Zotero. As the search was conducted in a single database, no true duplicate records were identified.

Title screening: All 3,841 records were screened at the title level. Studies were excluded if they addressed environmental or occupational exposure, diagnostic imaging (MRI safety), radiofrequency exposure, non-oncological populations, or purely physical or engineering outcomes. After title screening, 599 records remained.

Abstract screening: Abstracts of the 599 records were reviewed. Studies were excluded if they did not involve oncological patients, lacked therapeutic intent, or were purely preclinical. After abstract screening, 205 reports were assessed for eligibility and subsequently classified according to the predefined research questions (Q1-Q3). Only four studies specifically addressed oncological safety (Q1) and were therefore assessed at the full-text level for this objective.

Eligibility assessment and study selection: Eligible studies included randomized controlled trials, non-randomized interventional studies, and observational clinical studies involving oncological patients exposed to therapeutic magnetic fields. 

For the primary objective (Q1), only studies explicitly assessing oncological safety outcomes, such as disease progression, recurrence, survival, or response to oncological treatments, were eligible for full-text evaluation.

Four studies were assessed at the full-text level for Q1 (oncological safety). Three studies were excluded due to the absence of hard oncological endpoints or the unavailability of the full text. One randomized controlled trial was included in the final qualitative synthesis.

For secondary (Q2) and tertiary (Q3) objectives, studies addressing supportive care during oncological treatments or management of comorbidities were identified through narrative classification and were not subjected to formal full-text eligibility assessment.

With regard to supportive and adjunctive applications (Q2), several studies (approximately 15-20) were identified, mainly addressing chemotherapy-induced peripheral neuropathy (CIPN), treatment-related symptoms, and quality of life outcomes. A larger and more heterogeneous body of literature (approximately 20-30 studies) explored the management of musculoskeletal comorbidities in oncological patients (Q3), predominantly through observational, non-controlled, or narrative reports. These studies were identified through narrative classification and were not subjected to a formal full-text eligibility assessment; therefore, the reported numbers should be considered indicative.

Given the extremely limited number of studies eligible for the primary objective (Q1), with only one randomized controlled trial included in the final synthesis, and the substantial heterogeneity and narrative nature of studies addressing secondary and tertiary objectives (Q2-Q3), a formal risk of bias assessment was not performed.

Results

Oncological Safety of Therapeutic Magnetic Fields (Q1)

The characteristics and oncological outcomes of the study assessing oncological safety are summarized in Table 2.

**Table 2:** Studies assessing oncological safety outcomes of therapeutic magnetic fields (Q1).

Author (year)	Study design	Oncological population	Type of magnetic field	Intervention/Comparator	Oncological endpoints assessed	Main oncological results	Safety conclusions
Zhu et al. [25]	Randomized, double-blinded, placebo-controlled clinical trial	Patients with advanced non-small cell lung cancer (NSCLC)	Low-frequency rotating static magnetic field (0.4 T; 7.5 Hz)	Chemotherapy + magnetic field therapy vs chemotherapy + sham	Progression-free survival (PFS); Objective response rate (ORR); Disease control rate (DCR)	No significant differences were observed between groups in PFS, ORR, or DCR. Oncological outcomes were comparable between the experimental and control groups.	Exposure to therapeutic magnetic fields in combination with chemotherapy was not associated with worsening oncological outcomes under the studied conditions.

Supportive and Adjunctive Use of Therapeutic Magnetic Fields (Q2)

A total of three clinical studies [26-28] evaluated the use of therapeutic magnetic fields as a supportive or adjunctive intervention during oncological treatments (Table 3).

**Table 3:** Clinical studies assessing the supportive and adjunctive use of therapeutic magnetic fields in oncological patients (Q2). CIPN, Chemotherapy-Induced Peripheral Neuropathy; MFT, Magnetic Field Therapy; CTCAE, Common Terminology Criteria for Adverse Events; NCV, Nerve Conduction Velocity; EORTC QLQ-C30, European Organisation for Research and Treatment of Cancer Quality of Life Questionnaire Core 30

Author (year)	Design	Population	Magnetic field protocol	Comparator	Outcomes	Main findings
Geiger et al. [26]	Phase II pilot (pre-post)	Cancer patients with CIPN; n = 20	Low-frequency MFT 4-12 Hz, ~3-4 weeks	None	CTCAE neuropathy items; NCV	Improvement in sensory ataxia/neuropathy and neuropathic pain; NCV increase reported
Rick et al. [27]	Phase III RCT, double-blind, placebo-controlled	Histologically confirmed cancer + neurotoxic chemo history; n = 44	Hand-held device (MAGCELL MICROCIRC): rotating magnetic disc 4-12 Hz, 5-min cycles; surface flux density ~420 mT peak-to-peak; follow-up T1, T2 (3 wks), T3 (3 mos)	Placebo device	Primary: NCV. Secondary: CTCAE score; painDETECT end score	NCV improved at T3 in MFT (primary endpoint met; p = 0.015, especially peroneal sensory neurotoxicity). CTCAE improved vs placebo (p = 0.04). painDETECT, not significantly different (p = 0.11)
Koneva et al. [28]	Observational cohort study	Patients with melanoma, lung cancer, and renal cancer receiving immunotherapy; n = 48	Therapeutic magnetotherapy (protocol not fully standardized)	Immunotherapy alone	EORTC QLQ-C30; CTCAE	Improved global health status and quality of life; reduced treatment-related adverse events compared with controls

Most studies focused on CIPN, treatment-related symptoms, and quality of life. These studies reported significant improvements in neuropathic symptoms, pain intensity, and functional outcomes in patients receiving magnetic field therapy in addition to standard oncological care. Improvements in quality of life, assessed using validated instruments such as the EORTC QLQ-C30 (European Organisation for Research and Treatment of Cancer Quality of Life Questionnaire Core 30), were consistently observed across studies, particularly in patients undergoing chemotherapy or immunotherapy.

Importantly, none of the included studies reported an increase in treatment-related toxicity or a worsening of patients’ clinical status attributable to exposure to magnetic fields.

Two clinical studies evaluated low-frequency magnetotherapy in the treatment of CIPN.

In a phase II pilot trial (n = 20), Geiger et al. [26] reported an improvement in neuropathic symptoms (sensory ataxia, neuropathy, and neuropathic pain) after three to four weeks of treatment with low-frequency magnetic fields (4-12 Hz), accompanied by an increase in nerve conduction velocity (NCV).

A randomized phase III trial [27], double-blinded and placebo-controlled (n = 44), compared magnetotherapy using a dedicated device versus placebo, with assessments performed at baseline (T1), three weeks (T2), and three months (T3). The primary endpoint (improvement in NCV at T3) was statistically significant in the magnetotherapy group (p = 0.015), particularly for sensory neurotoxicity of the peroneal nerve. Among secondary endpoints, a significant improvement in perceived neurotoxicity assessed by CTCAE was observed in favor of the magnetotherapy group (p = 0.04), whereas neuropathic pain measured by the painDETECT end score did not differ significantly between groups (p = 0.11).

In addition, one observational clinical study evaluated the use of therapeutic magnetic fields as a supportive intervention during immunotherapy. In a cohort study involving patients with melanoma, lung cancer, and renal cancer, Koneva et al. [28] reported improvements in global health status and quality of life, assessed by the EORTC QLQ-C30, along with a reduction in treatment-related adverse events, evaluated according to CTCAE criteria, compared with control groups receiving immunotherapy alone.

Management of Musculoskeletal Comorbidities in Oncological Patients (Q3)

No clinical studies specifically designed to evaluate the effectiveness of therapeutic magnetic fields for the treatment of musculoskeletal comorbidities, such as low back pain or osteoarthritis, in oncological populations were identified.

Evidence relevant to this research question was limited to studies conducted in non-oncological populations, which reported beneficial effects of magnetic field therapies on pain reduction and functional improvement in musculoskeletal disorders. However, these findings could not be directly extrapolated to oncological patients. Therefore, no quantitative or qualitative synthesis of clinical outcomes for Q3 in oncological populations was possible.

Discussion

The discussion is structured according to the three predefined research questions (Q1-Q3), in line with the organization of the Results section. Specifically, we first address the issue of oncological safety of therapeutic magnetic fields (Q1), followed by their potential role as supportive or adjunctive interventions during oncological treatments (Q2), and finally, their possible application in the management of musculoskeletal comorbidities in oncological patients (Q3).

Oncological Safety of Therapeutic Magnetic Fields (Q1)

The use of therapeutic magnetic fields in oncological patients has historically been regarded with caution in daily clinical practice and is often considered contraindicated, particularly in the presence of active or metastatic disease.

This caution largely stems from the lack of robust evidence, and from a precautionary interpretation of the potential biological effects of physical fields on rapidly proliferating tissues, rather than from clear clinical data demonstrating a direct oncogenic effect. Consequently, many clinical sources and practical guidelines in physical therapy and integrative medicine discourage the unsupervised use of magnetotherapy in patients with cancer or active malignancy, or recommend considering it only under strict medical supervision and in the absence of more established alternatives [22,29]. In the field of integrative oncology, recognized institutions such as the Memorial Sloan Kettering Cancer Center highlight that, while some physical therapies may be integrated into cancer care pathways, the use of magnetotherapy is not supported by sufficient evidence to be recommended as an oncological treatment, and its use is therefore advised with caution, particularly in cases of active cancer [22]. Similarly, educational resources in physical therapy and physiotherapy report that active neoplastic conditions are traditionally considered among the precautions or contraindications for the application of many physical modalities, including magnetic fields, without specifying a clearly defined pathophysiological mechanism [29].

It should be emphasized that these recommendations primarily reflect precautionary clinical attitudes and expert consensus rather than direct empirical evidence of oncological harm. Accordingly, such sources are cited to contextualize prevailing clinical practice, while conclusions regarding oncological safety in the present review are derived exclusively from empirical clinical studies explicitly assessing oncological outcomes and supported by experimental evidence for biological plausibility.

These pragmatic indications, therefore, reflect a conservative clinical approach adopted in the absence of robust evidence clearly defining oncological safety, and underscore the need for controlled clinical studies to guide informed therapeutic decisions in this patient population.

However, unlike ionizing radiation, low-intensity and/or low-frequency magnetic fields do not induce direct DNA damage and are not associated with recognized mutagenic mechanisms [2].

Additional preclinical evidence from animal models suggests that low-frequency magnetic fields, such as rotating magnetic fields, do not exhibit adverse effects on oncological processes and, under certain experimental conditions, may even be associated with reduced tumor growth compared with controls [30]. These data provide a broader biological context for understanding the interactions between magnetic fields and neoplastic tissue, although they are not directly transferable to clinical practice in oncological patients.

Concurrently, experimental and preliminary clinical studies have explored the interaction of low-frequency magnetic fields with biological systems, suggesting predominantly non-thermal effects and modulation of specific biochemical reactions and cellular signaling pathways potentially relevant to antitumor activity [19,30-33].

In this context, the results of the present systematic review highlight a marked discrepancy between widespread clinical caution and the actual availability of controlled clinical evidence. With regard to oncological safety (Q1), only one randomized clinical trial explicitly evaluated “hard” oncological endpoints in patients exposed to therapeutic magnetic fields in combination with standard antineoplastic treatments. In the study by Zhu et al. [25], conducted in patients with advanced non-small cell lung cancer undergoing chemotherapy, the addition of a low-frequency rotating static magnetic field was not associated with worsening of progression-free survival, objective response rate, or disease control rate compared with sham treatment.

Although these findings do not allow definitive conclusions regarding long-term oncological safety, they do not support the hypothesis of a direct oncogenic effect or a negative interference with the efficacy of antineoplastic treatments under the studied conditions. It is, nevertheless, important to emphasize that the scarcity of clinical studies with primary oncological endpoints represents a major limitation of the available evidence. The absence of signals of harm should not be interpreted as definitive proof of safety, but rather as an indication that oncological safety has rarely been investigated as a primary outcome.

In parallel, several preclinical studies have explored the interaction between low-frequency magnetic fields and neoplastic tissue, suggesting predominantly non-thermal effects and modulation of specific cellular pathways involved in proliferation, apoptosis, oxidative stress, and inflammatory signaling [19,31,34,35]. These studies do not demonstrate consistent oncogenic effects and, in some experimental models, even suggest inhibition of tumor growth.

However, such evidence remains confined to the experimental setting and cannot be directly translated into clinical practice.

Therapeutic Magnetic Fields as Supportive Interventions During Oncological Treatments (Q2)

Chemotherapy-induced peripheral neuropathy (CIPN) (Q2.1): CIPN represents one of the most frequent and disabling complications of systemic oncological treatments, with a significant impact on quality of life and treatment adherence. In this context, low-frequency magnetotherapy (a form of PEMF) has been investigated in a limited number of prospective clinical studies.

In a phase II pilot trial, Geiger et al. [26] reported an improvement in neuropathic symptoms and an increase in NCV after several weeks of treatment with low-frequency magnetic fields. These observations were partially confirmed by a randomized, double-blind, placebo-controlled phase III study conducted by Rick et al. [27], which demonstrated a significant improvement in NCV at three months in the magnetotherapy group, along with a reduction in neurotoxicity assessed according to the Common Terminology Criteria for Adverse Events (CTCAE). The CTCAE is a standardized ordinal scale widely used in oncological trials to classify the severity of adverse events; within this system, sensory peripheral neuropathy is graded from 1 to 4 based on symptom intensity and the degree of interference with activities of daily living [36].

However, the absence of significant differences in neuropathic pain and the improvement also observed in the placebo group suggest that the effect of magnetotherapy may be more pronounced on neurophysiological and sensory aspects rather than on the algic component of neuropathy. In addition, the limited sample size and single-center design of the studies warrant a cautious interpretation of the results.

Quality of life and tolerability of oncological treatments (Q2.2): Preservation of quality of life and treatment tolerability represents a central objective in the management of oncological patients, particularly in cases of advanced disease. In this context, one clinical study evaluated the effect of magnetotherapy as a supportive intervention during immunotherapy.

In the study by Koneva et al. [28], conducted in patients with melanoma, lung cancer, and renal cancer, the addition of magnetotherapy was associated with a significant improvement in global quality of life, assessed using the EORTC QLQ-C30 questionnaire [37], as well as a reduction in the severity of adverse events evaluated according to the CTCAE version 5.0 criteria [38]. Although these findings are clinically relevant, the lack of formal randomization, the small sample size, and the single-center nature of the study limit the generalizability of the conclusions.

Other clinical evidence and grey literature: Beyond the controlled clinical studies included in the present systematic review, the literature reports numerous clinical experiences on the use of magnetotherapy in oncology, predominantly originating from Eastern European settings and published in local journals or in sources not indexed in major international biomedical databases.

Another relevant line of investigation concerns the rehabilitation of patients undergoing radical hysterectomy [39] and radical mastectomy [25,40-42], as well as those with ovarian cancer [43] and bone sarcoma [44]. Several of these studies, conducted in Russia and other Eastern European countries, report the use of magnetotherapy - often in combination with other physical modalities (e.g., intermittent pneumocompression and/or radiotherapy) - for the treatment of pain, lymphedema, and upper limb functional limitations [40-42,45]. Although these studies suggest potential clinical benefits, their methodological heterogeneity and the frequent unavailability of full texts limit their inclusion in a quantitative systematic synthesis.

Finally, experiences with the use of magnetotherapy in oncological rehabilitation programs in patients in remission have been reported [40,45], including pediatric populations with neurological comorbidities [46,47]. In these cases as well, the available data are predominantly observational and uncontrolled, but they contribute to documenting a relatively widespread clinical use of magnetotherapy in oncological contexts, in the absence of systematic reports of oncological adverse events [30].

Overall, this grey and non-indexed literature suggests that the clinical use of magnetotherapy in oncology is broader than what emerges from the available controlled studies. However, the methodological limitations of these works preclude definitive conclusions regarding both efficacy and oncological safety, reinforcing the need for adequately designed prospective randomized studies.

In the absence of robust clinical evidence, several preclinical (in vitro) studies have explored the effects of magnetic fields on tumor cell models, assessing their impact on proliferation [48], apoptosis [49-53], inhibition of growth and metastasis [54-57], and inflammatory pathways [58]. Experimental in vitro studies have also suggested that exposure to magnetic fields may modulate tumor cell responses to chemotherapeutic agents [59]. In particular, Chakkalakal et al. [60] observed an inhibition of proliferation in human osteosarcoma cells treated with adriamycin in the presence of magnetic fields, suggesting a possible non-thermal biological interaction between magnetic fields and cytotoxic treatments.

These studies have not provided consistent evidence of a direct pro-oncogenic effect but remain confined to the experimental setting and do not allow direct clinical extrapolation [19].

This leads to an evident paradox: on the one hand, the clinical use of magnetotherapy in oncological and rehabilitative settings is reported in numerous clinical experiences; on the other hand, the available controlled clinical literature is limited and fragmented, and does not definitively clarify either the oncological safety or the therapeutic efficacy of such interventions. In this context, the persistence of a generalized contraindication to the use of magnetic fields in oncological patients appears not to be adequately supported by robust clinical evidence.

Cancer-related pain and palliation (Q2.3): Cancer-related pain and associated symptoms represent a substantial component of disease burden, particularly in patients with advanced or metastatic malignancies, with a significant impact on quality of life and clinical course [45,61]. The available literature on the use of magnetotherapy in palliative settings consists predominantly of observational studies, case series, and clinical reports. These works describe subjective improvements in pain intensity and general well-being, for example, in pain related to bone metastases or in post-oncological rehabilitation contexts [45,61]. However, methodological weaknesses, the absence of control groups, and the predominantly subjective nature of the outcomes render this evidence exploratory and hypothesis-generating rather than conclusive [30].

In this context, magnetotherapy may be considered, at most, as a potentially safe complementary intervention to be used in selected settings and under clinical supervision.

Management of Comorbidities in Oncological Patients (Q3)

Oncological patients frequently present with musculoskeletal and neurological comorbidities, such as low back pain, osteoarthritis, and functional disorders, which may further compromise quality of life and overall functional status [62]. Although no clinical studies have been specifically designed to evaluate the effectiveness of magnetotherapy in the treatment of these comorbidities in oncological populations, numerous studies conducted in non-oncological populations have demonstrated a potential benefit of magnetic fields in the management of low back pain [4-6,63] and degenerative joint diseases [7].

It should also be considered that the prevailing tendency to avoid magnetotherapy, not only in patients with active malignancy but also in those in remission or even in long-term cancer survivors, may have unintended negative consequences. In the management of musculoskeletal conditions, such as spinal stenosis or advanced hip and knee osteoarthritis, alternative pharmacological or surgical treatments may carry a substantially higher risk of complications, particularly in frail or multimorbid oncological patients.

In this context, a solely precautionary approach, based on theoretical concerns rather than documented oncological harm, may not necessarily represent the most beneficial option for the patient. While caution remains appropriate in the absence of robust safety data, an indiscriminate avoidance of non-ionizing physical therapies, such as magnetotherapy, could potentially limit access to conservative treatment options that are otherwise associated with a favorable safety profile. This consideration further highlights the need for balanced, evidence-informed decision-making and for dedicated clinical studies addressing the safety and effectiveness of magnetotherapy in oncological populations across different phases of disease and survivorship.

In the absence of documented evidence of oncological harm, and considering the available biological and clinical rationale, magnetotherapy could be hypothesized as a therapeutic option for the management of musculoskeletal comorbidities in selected oncological patients. However, this hypothesis remains indirect and requires confirmation through dedicated clinical studies.

Several methodological limitations should be acknowledged when interpreting the findings of this review. In particular, the absence of a formal risk-of-bias assessment reflects the extremely limited number of clinical studies addressing oncological safety outcomes and the marked heterogeneity of the available literature, rather than a methodological oversight. Only one randomized controlled trial was eligible for full-text inclusion for the primary outcome (Q1), precluding meaningful application of standardized risk-of-bias tools. For secondary and tertiary objectives (Q2-Q3), studies were identified through narrative classification and were not suitable for structured bias assessment. These limitations underscore the need for well-designed prospective trials specifically addressing oncological safety and supportive outcomes of therapeutic magnetic fields.

At present, no standardized oncological standard operating procedures (SOPs) or formal clinical guidelines specifically address the use of therapeutic magnetic fields in cancer patients; their role is only marginally mentioned within broader reviews on oncological therapeutic strategies and innovations [64].

Clinical implications and future perspectives

Overall, an evident paradox emerges: on the one hand, a non-negligible clinical use of magnetotherapy in oncological patients; on the other hand, a scarce and fragmented body of controlled clinical literature that does not allow definitive clarification of either oncological safety or the therapeutic efficacy of these interventions. In this context, the persistence of a generalized contraindication to the use of magnetic fields in oncological patients appears to be inadequately supported by the currently available clinical evidence, and underscores the need for prospective, controlled, and adequately designed clinical studies to guide evidence-based therapeutic decision-making.

## Conclusions

This systematic review highlights a substantial gap between the widespread clinical caution surrounding therapeutic magnetic fields in oncology and the limited availability of clinical evidence supporting such contraindications. Only one randomized controlled trial directly evaluated oncological safety and did not demonstrate adverse oncological outcomes associated with magnetic field exposure. In contrast, a broader body of literature suggests potential benefits of magnetic fields as supportive interventions for symptom management, quality of life, and rehabilitation in oncological patients. Although current evidence remains heterogeneous and largely exploratory, it does not indicate oncological harm, and further well-designed clinical trials are warranted to clarify the role of therapeutic magnetic fields in oncological care.
